# Highly Resolved Papilionoid Legume Phylogeny Based on Plastid Phylogenomics

**DOI:** 10.3389/fpls.2022.823190

**Published:** 2022-02-23

**Authors:** In-Su Choi, Domingos Cardoso, Luciano P. de Queiroz, Haroldo C. de Lima, Chaehee Lee, Tracey A. Ruhlman, Robert K. Jansen, Martin F. Wojciechowski

**Affiliations:** ^1^School of Life Sciences, Arizona State University, Tempe, AZ, United States; ^2^National Institute of Science and Technology in Interdisciplinary and Transdisciplinary Studies in Ecology and Evolution (INCT IN-TREE), Instituto de Biologia, Universidade Federal da Bahia, Salvador, Brazil; ^3^Department of Biological Sciences, Universidade Estadual de Feira de Santana, Feira de Santana, Brazil; ^4^Instituto de Pesquisas Jardim Botânico do Rio de Janeiro, Rio de Janeiro, Brazil; ^5^Department of Integrative Biology, University of Texas at Austin, Austin, TX, United States; ^6^Center of Excellence for Bionanoscience Research, King Abdulaziz University (KAU), Jeddah, Saudi Arabia

**Keywords:** deep evolution, Meso-Papilionoideae, plastid genome, Papilionoideae, Leguminosae

## Abstract

Comprising 501 genera and around 14,000 species, Papilionoideae is not only the largest subfamily of Fabaceae (Leguminosae; legumes), but also one of the most extraordinarily diverse clades among angiosperms. Papilionoids are a major source of food and forage, are ecologically successful in all major biomes, and display dramatic variation in both floral architecture and plastid genome (plastome) structure. Plastid DNA-based phylogenetic analyses have greatly improved our understanding of relationships among the major groups of Papilionoideae, yet the backbone of the subfamily phylogeny remains unresolved. In this study, we sequenced and assembled 39 new plastomes that are covering key genera representing the morphological diversity in the subfamily. From 244 total taxa, we produced eight datasets for maximum likelihood (ML) analyses based on entire plastomes and/or concatenated sequences of 77 protein-coding sequences (CDS) and two datasets for multispecies coalescent (MSC) analyses based on individual gene trees. We additionally produced a combined nucleotide dataset comprising CDS plus *matK* gene sequences only, in which most papilionoid genera were sampled. A ML tree based on the entire plastome maximally supported all of the deep and most recent divergences of papilionoids (223 out of 236 nodes). The Swartzieae, ADA (Angylocalyceae, Dipterygeae, and Amburaneae), Cladrastis, Andira, and Exostyleae clades formed a grade to the remainder of the Papilionoideae, concordant with nine ML and two MSC trees. Phylogenetic relationships among the remaining five papilionoid lineages (Vataireoid, *Dermatophyllum*, Genistoid s.l., Dalbergioid s.l., and Baphieae + Non-Protein Amino Acid Accumulating or NPAAA clade) remained uncertain, because of insufficient support and/or conflicting relationships among trees. Our study fully resolved most of the deep nodes of Papilionoideae, however, some relationships require further exploration. More genome-scale data and rigorous analyses are needed to disentangle phylogenetic relationships among the five remaining lineages.

## Introduction

Advances in next-generation sequencing and computational resources have enabled unparalleled phylogenomic analyses. These studies have deepened our understanding of evolutionary relationships across many branches in the plant Tree of Life, from the most recalcitrant deep relationships at and within the family level (e.g., Xi et al., 2012; Goremykin et al., 2015; Duvall et al., 2020; Koenen et al., 2020a; Yang et al., 2020; Antonelli et al., 2021; Orton et al., 2021; Schneider et al., 2021; Serna-Sánchez et al., 2021) to long, unresolved radiations at the species level (e.g., Nicholls et al., 2015; Welch et al., 2016; Villaverde et al., 2018; Thode et al., 2020; Pereira et al., 2021). While massive amounts of plastid genome (plastome) sequence data have filled the family level sampling gap for angiosperms (e.g., Li et al., 2021), infra-family levels remain less well covered. This is particularly true of the economically important, ecologically successful, morphologically diverse, species-rich legume family Fabaceae (Leguminosae), from which the plastomes of only 319 species in 184 genera have been deposited in the GenBank database^1^ (Accessed Sep. 09, 2021) thus far, of the more than 22,000 species and 770 genera in six subfamilies (LPWG, 2017, 2021).

Fabaceae is one of the most spectacular examples of diversification among flowering plants. Many legumes are not only ecologically dominant across major tropical and subtropical biomes (Schrire et al., 2005; DRYFLOR, 2016; LPWG, 2017) but symbiotically associated with nitrogen-fixing bacteria *via* root nodules (Sprent et al., 2017), amplifying the family’s importance for food security, sustainable agriculture, and ecosystem function (Food and Agriculture Organization^2^; LPWG, 2013; Yahara et al., 2013). The successful radiation of legumes is thought to be associated with plant defense strategies against herbivores, diverse, intimate ecological interactions involving ant-housing domatia and ant-feeding extrafloral nectaries (Janzen, 1966; Marazzi and Sanderson, 2010; Chomicki et al., 2015; Marazzi et al., 2019), an extraordinary range of floral forms (Tucker, 2003a) and pollination mechanisms (Arroyo, 1981). The family provides a wide diversity of secondary metabolites (alkaloids, flavonoids, lignans, tannins, terpenoids, benzofuranoids, and non-proteinogenic amino acids such as canavanine; Bisby et al., 1994; Kursar et al., 2009; Wink, 2013). The family is also an excellent model to reveal the patterns and processes of plastome structural evolution, since its taxa have undergone several dramatic rearrangements involving inversions of large blocks of sequence, contraction, loss, and regain of the inverted repeat (IR), gene/intron loss and repeat accumulation (e.g., Palmer et al., 1987; Lavin et al., 1990; Doyle et al., 1996; Jansen et al., 2008; Martin et al., 2014; Schwarz et al., 2015; Choi and Choi, 2017; Choi et al., 2019; Zhang et al., 2020; Charboneau et al., 2021; Lee et al., 2021). One of the most striking examples is a 50-kb inversion situated in the plastome large single-copy region (LSC) that is shared by the vast majority of subfamily Papilionoideae (papilionoids) (Doyle et al., 1996; Pennington et al., 2001; Wojciechowski et al., 2004; Cardoso et al., 2012a; LPWG, 2013). The 50 kb-inversion was long considered an unequivocal molecular synapomorphy for this clade, but recently at least three species of *Sesbania* Adans. were shown to have completely reverted the 50-kb sequence, resulting in essentially the same gene order as found in the earliest-diverging papilionoids (Lee et al., 2021).

In addition to providing a model for plastome structural rearrangements, Papilionoideae, the largest legume subfamily with an estimated 501 genera and 14,000 species (LPWG, 2021), also exhibits an impressive morphological diversity (Lewis et al., 2005; LPWG, 2017). For example, the early diversification of the Papilionoideae is marked by multiple evolutionary shifts in floral architecture (Ireland et al., 2000; Pennington et al., 2001; Cardoso et al., 2012a, 2013a,b; Klitgård et al., 2013; Ramos et al., 2016; Castellanos et al., 2017). Genera that were traditionally classified in the “primitive” tribes Sophoreae and Swartzieae (e.g., Cowan, 1981; Polhill, 1981a) are now phylogenetically scattered among the early-branching lineages of Papilionoideae. Their flowers are morphologically variable, from actinomorphic (radial or polysymmetric) with five undifferentiated petals to zygomorphic (bilateral or monosymmetric) with the petals poorly differentiated, but also absent or restricted to just the adaxial standard petal, and with free, often numerous stamens (Pennington et al., 2000; Cardoso et al., 2012a). The main phylogenetic outcome from early, single molecular locus Papilionoideae phylogenies (Doyle et al., 1997; Pennington et al., 2001; Wojciechowski et al., 2004) was that the papilionate-flowered ancestors experienced high evolutionary lability during the initial diversification of the subfamily, refuting the notion that non-papilionate flowers represented signatures of antiquity (e.g., Arroyo, 1981; Polhill, 1981a; Tucker and Douglas, 1994).

The extraordinary evolutionary and ecological success of legumes during their more than 60 million years of diversification history (Lavin et al., 2005; Bruneau et al., 2008; Koenen et al., 2021) may be related to the macroevolutionary stability of the highly specialized papilionate flower (Cardoso et al., 2013a), beneficial associations with nodulating symbiotic bacteria (Sprent et al., 2017), ant feeding *via* extrafloral nectaries (Marazzi et al., 2012, 2019) and/or secondary metabolite accumulation (Wink, 2013). Determining the emergence and influence of these phenomena in the evolutionary history of legumes requires a well-sampled and fully resolved phylogeny.

Previous phylogenetic studies using the coding sequences of the plastid *rbcL* (e.g., Doyle et al., 1997; Kajita et al., 2001) and *matK* genes (e.g., Wojciechowski et al., 2004; Cardoso et al., 2012a, 2013a, 2015; Ramos et al., 2016; Castellanos et al., 2017; LPWG, 2017; Queiroz et al., 2017), as well as a supermatrix approach (McMahon and Sanderson, 2006), sampled densely across the Papilionoideae, revealed many new clades, unexpected generic re-alignments, and placed several taxonomically orphan genera. However, these studies left the Papilionoideae backbone phylogeny and the placement of several evolutionary key genera largely unresolved. Such is the case with the small temperate North American genus *Dermatophyllum* Scheele. Apart from the Genistoid s.l. clade, *Dermatophyllum* is the only lineage of legumes known to accumulate a variety of quinolizidine alkaloids (QA), a class of alkaloids mainly distributed in these two papilionoid lineages with some phylogenetically scattered occurrences within other angiosperm families (Bisby et al., 1994; Kite and Pennington, 2003; Lee et al., 2013; Wink, 2013). Determining the sister relationship of this enigmatic, isolated genus with respect to the genistoids is fundamental to answer whether the evolution of such an important secondary metabolite in papilionoids had a single or multiple origins. Also unresolved is the polytomy within the large 50-kb-inversion clade involving the species-rich Dalbergioid s.l. and Non-Protein Amino Acid Accumulating (NPAAA) clades, as well as the florally heterogeneous Andira, Exostyleae, and Vataireoid clades. Previous phylogenies with broad taxon sampling resolved the earliest divergences of the Papilionoideae involving the swartzioids and the ADA clade (Angylocalyceae, Dipterygeae, and Amburaneae), albeit with low support values. These clades are often interchangeably shown as sister to the remainder of the subfamily (Cardoso et al., 2012a, 2013a, 2015; Ramos et al., 2016; Zhang et al., 2020; Zhao et al., 2021).

Recent advances in deep- and genus-level legume phylogenomics using both plastid and nuclear genes (Cannon et al., 2015; LPWG, 2017; Vatanparast et al., 2018; Ojeda et al., 2019; Koenen et al., 2020a,b, 2021; Oyebanji et al., 2020; Zhang et al., 2020; Zhao et al., 2021) have contributed to the resolution of some of the obscure deep relationships in the Papilionoideae. Still, many morphological key genera in the early-diverging lineages outside the agriculturally important NPAAA clade (Cardoso et al., 2012a, 2013a) have not been evaluated in any phylogenomic study. Among the legume phylogenomic analyses that more thoroughly investigated the Papilionoideae (Koenen et al., 2020a; Zhang et al., 2020; Zhao et al., 2021), only Zhao et al. (2021) have greatly improved taxon sampling within the early-diverging clades outside the NPAAA clade. In that study, more than 1,500 nuclear genes from transcriptome and genome assemblies of 217 papilionoid genera were explored. Previous plastome-inferred legume phylogenies have sampled just 32 (Koenen et al., 2020a) and 48 (Zhang et al., 2020) papilionoid genera. These studies all left important gaps, for example, regarding the placement of *Dermatophyllum*, either because no representative species were sampled or because statistical support for its sister relationship was relatively low. Thus, we still lack a clearer, more focused picture of Papilionoideae evolutionary history from comprehensively sampled plastome data.

In this study, we used a denser taxon sampling of plastome sequence data with a focus on the early-branching Papilionoideae. Despite the fact that plastome data may produce conflicting topologies (e.g., Gonçalves et al., 2019; Walker et al., 2019; Koenen et al., 2020a), examining them thoroughly can help to clarify the phylogenetic signal in concatenated analyses of a large number of plastid loci (Gonçalves et al., 2019; Zhang et al., 2020). For example, the inclusion of protein-coding genes only, removal of ambiguously aligned regions, or the use of coalescent methods (e.g., Gonçalves et al., 2019) may be helpful to arrive at phylogenetically accurate topologies. Here, we aimed to explore phylogenetic signals across diverse plastome-derived datasets together with cross-genomic comparisons of mitochondrial and nuclear data and shed light on key evolutionary relationships related to a number of questions that have persisted in the subfamily.

## Materials and Methods

### Plant Material Sampling

Our taxon sampling strategy was designed to cover the major papilionoid clades recognized by Cardoso et al. (2012a, 2013a), especially those in need of additional investigation. Thirty-eight taxa were collected from Brazil and the United States of America. Seeds of *Phaseolus acutifolius* A. Gray were acquired from the Desert Legume Program (University of Arizona, Tucson) and grown in the University of Texas at Austin (UT-Austin) greenhouse. Leaf samples were collected, flash-frozen in liquid nitrogen and stored at −80°C before DNA isolation except the silica-gel dried leaf tissues of *Astragalus canadensis* var. *brevidens* (Gand.) Barneby. Voucher specimens are housed at the Billie L. Turner Plant Resource Center at UT-Austin (TEX-LL), the Arizona State University (ASU), the Universidade Estadual de Feira de Santana (HUEFS), and the Jardim Botânico do Rio de Janeiro (RB) herbaria. Collection information for a total of 39 taxa is available in Supplementary Table 1.

### Next-Generation Sequencing and Plastid Genome Completion

Total genomic DNAs were extracted by the hexadecyltrimethylammonium bromide protocol, described in Doyle and Doyle (1987), or using the NucleoSpin Plant II, Mini Kit for DNA from plants (Macherey-Nagel, Düren, Germany). Next-generation sequencing (NGS) reads (2 × 150 bp) from ca. 300 bp insert libraries for 38 taxa were generated by the Beijing Genomics Institute (BGI; Shenzhen, China) using the MGI DNBseq™ platform (MGI Tech Co., Shenzhen, China). Total genomic DNA of *A. canadensis* var. *brevidens* was sequenced using the Illumina NextSeq 500 at the Arizona State University Genomics Facility (2 × 151 bp; ca. 300 bp insert size). Plastomes were assembled and annotated by the methods described in Choi et al. (2019).

### Plastid Phylogenomics

In addition to the complete plastomes generated in this study, we obtained the plastome sequence from at least a single species of each papilionoid genus currently available in GenBank (see text footnote 1) (Accessed May 24, 2021) or Dryad^3^ (Accessed Aug. 24, 2020). In total, 244 taxa, with 237 papilionoids (174 genera) and seven non-papilionoids were collected as ingroup and outgroup, respectively (Supplementary Table 2). We produced nine sequence alignments as data sets for maximum likelihood (ML) analysis and two gene tree sets for species tree estimation based on the multispecies coalescent (MSC) approach (Table 1 and Supplementary Table 3).

**TABLE 1:** Descriptions for 11 datasets used for plastid phylogenomic analyses in this study.

Tree number	Dataset name	Description
1	WP	Whole plastid genome alignment
2	WP_nogap	WP dataset with all indels removed
3	WP_gb	WP dataset with only conserved sequence blocks
4	CD_nt	Concatenation of 77 CDS nucleotide alignments
5	CD_nt_gb	CD_nt dataset with only conserved sequence blocks
6	CD_nt_dg	CD_nt dataset with degenerated synonymous substitutions sites
7	CD_aa	Concatenation of 77 CDS amino acid alignments
8	CD_aa_gb	CD_aa dataset with only conserved sequence blocks
9	CD_matK_cb	Combined dataset of CD_nt and *matK*-only dataset
10	77 gene trees	All 77 individual gene trees
11	26 gene trees	26 gene trees with a mean bootstrap value higher than 85

Datasets 1—3 (WP, WP_nogap, and WP_gb; see Table 1) were prepared based on whole plastome alignments. For the legume taxa with two copies of the large IR, one copy was deleted. To align plastomes with different gene order, Mauve 2.3.1 (Darling et al., 2010) was used to detect locally colinear blocks (LCBs) relative to *Cercis canadensis* L. (KF856619). Sequence blocks of all taxa were rearranged to be colinear with *C. canadensis*. To avoid introducing non-orthologous sequences during the rearrangements, non-genic edges of LCBs were deleted. The intergenic regions that coincide with the end points of the 50-kb inversion and an adjacent gene encoding *rps16*, pseudogenized in many papilionoids (Schwarz et al., 2015; Choi and Choi, 2017; Lee et al., 2021), were deleted from all taxa. Complete plastomes were aligned by MAFFT v7.450 (Katoh and Standley, 2013) in Geneious Prime 2021.0.3^4^ using default options. This raw alignment is designated as WP (dataset 1). The WP dataset was further refined by two different strategies. The WP_nogap (dataset 2) was prepared by deleting all indel regions using the “mask alignment” tool in Geneious Prime, as described in Orton et al. (2021). The WP_gb (dataset 3) was prepared from the WP dataset using Gblocks 0.91b (Castresana, 2000) using default options allowing selection of conserved sequence blocks without indel regions exclusively.

Datasets 4—8 (CD_nt, CD_nt_gb, CD_nt_dg, CD_aa, and CD_aa_gb; see Table 1) were prepared based on the protein-coding sequences (CDS) for 77 genes (Supplementary Table 4) extracted from each plastome. The 77 CDS included putatively pseudogenized genes with few mutations that could represent heteroplasmic variations (Park et al., 2020). In plastomes where the intactness of *rps16* was questionable, the pseudogene was unsampled. A single nucleotide (A, T, C, G, or N) was introduced or deleted to fit the reading frame when a gene included a premature stop codon due to indel polymorphism. Nucleotide sequences of each CDS dataset were aligned by MAFFT using the translation align option available in Geneious Prime. Finally, poorly aligned regions of each CDS alignment were manually adjusted or deleted. A concatenated aligned matrix of 77 CDS sequences was produced using the R package catGenes^5^. This concatenated nucleotide alignment (dataset 4) was designated CD_nt. This data set was further refined by Gblocks using default options resulting in CD_nt_gb (dataset 5). To avoid the problem of nucleotide compositional heterogeneity among taxa, the synonymous substitution sites in the CD_nt (dataset 4) were degenerated using Degen ver. 1.4.^6^ (Regier et al., 2010) and the result was designated CD_nt_dg (dataset 6). Datasets 7 and 8 were prepared based on translated amino acid sequences (AA) of CD_nt (dataset 4): Dataset 7 (CD_aa) was aligned without trimming and dataset 8 (CD_aa_gb) was trimmed using Gblocks with default options.

To more broadly examine the phylogenetic relationships of papilionoid legumes we included taxa that were not sampled in our plastome datasets but were previously sequenced for *matK*. We produced dataset 9 (CD_matK_cb; see Table 1) by combining CD_nt (dataset 4) and a *matK*-only dataset of 534 nucleotide sequences. For this alignment the taxa lacking plastome-scale data were represented by the *matK* coding region while their 76 CDS were coded as missing data. In total, dataset 9 included 771 papilionoids (478 genera) (Supplementary Table 5). The dataset included *matK* sequences used in LPWG (2017) and excluded duplications and short sequence fragments (< 800 bp).

For each dataset (1—9), ML analysis was conducted using IQ-TREE 1.6.12 (Nguyen et al., 2015) with 1,000 bootstrap (BS) replications, and a best-fit nucleotide substitution model was automatically selected based on the Bayesian information criterion.

Datasets 10 and 11 (see Table 1) consisted of sets of individual ML trees based on each CDS for species tree estimation with MSC approach using ASTRAL-III (Zhang et al., 2018). The ML analyses for each CDS were conducted as described above. To decrease error rate, branches with BS value lower than 10 were contracted as suggested in Zhang et al. (2018). Dataset 10 included all 77 individual trees. The individual trees with strong phylogenetic signals (average BS value > 85) were selected and this set of trees was designated dataset 11. The local posterior probability (LPP) and quartet score (QS) were calculated for all nodes.

Visualization and editing of phylogenetic trees were conducted using Interactive Tree Of Life (iTOL; Letunic and Bork, 2021), ggtree package (Yu et al., 2017), and custom R scripts.

## Results

**FIGURE 1:**
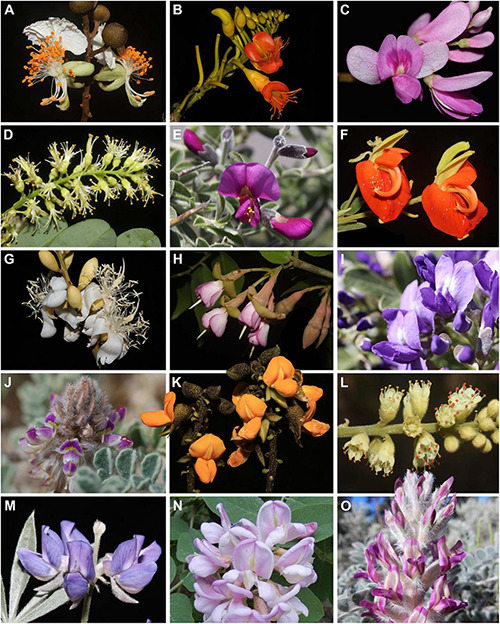
Broad variation in floral architecture across the papilionoid legumes (Papilionoideae). The selected taxa represent genera from the early-branching lineages **(A–M)** outside the NPAAA (non-protein amino acid accumulating) clade. **(A)** *Swartzia acutifolia* (Swartzieae); **(B)** *Castanospermum australe* [Angylocalyceae, ADA clade (Angylocalyceae, Dipterygeae, and Amburaneae)]; **(C)** *Dipteryx magnifica* (Dipterygeae, ADA clade); **(D)** *Myrocarpus fastigiatus* (Amburaneae, ADA clade); **(E)** *Pickeringia montana* (Cladrastis clade); **(F)** *Harpalyce brasiliana* (Brongniartieae, Genistoid s.l.); **(G)** *Aldina latifolia* (Andira clade); **(H)** *Exostyles venusta* (Exostyleae); **(I)** *Dermatophyllum secundiflorum* (unresolved); **(J)** *Dalea mollis* (Amorpheae); **(K)** *Centrolobium microchaete* (Dalbergieae); **(L)** *Leptolobium dasycarpum* (Leptolobieae, Genistoid s.l.); **(M)** *Lupinus sericeus* (Genisteae, Genistoid s.l.); **(N)** *Robinia neomexicana* (Robinieae, NPAAA); **(O)** *Astragalus mollissimus* (Astragalean, NPAAA); Photos by Domingos Cardoso **(A–D,F–H,K–M)** and Martin F. Wojciechowski **(E,I,J,N,O)**.

In total, we included plastomes from 244 species (Supplementary Table 2), 39 of which were sequenced for this study (Supplementary Table 1), and *matK* sequences from 534 species (Supplementary Table 5). This taxon sampling scheme includes representatives of all 22 main, early-diverging lineages (19 in plastome-only sampling, without *Amphimas* Pierre ex Harms, *Aldina* Endl., and *Clathrotropis macrocarpa* Ducke), as recognized by Cardoso et al. (2012a), and spans a diversity of taxa exhibiting radially symmetrical to bilaterally symmetrical floral architecture (Figure 1).

**FIGURE 2:**
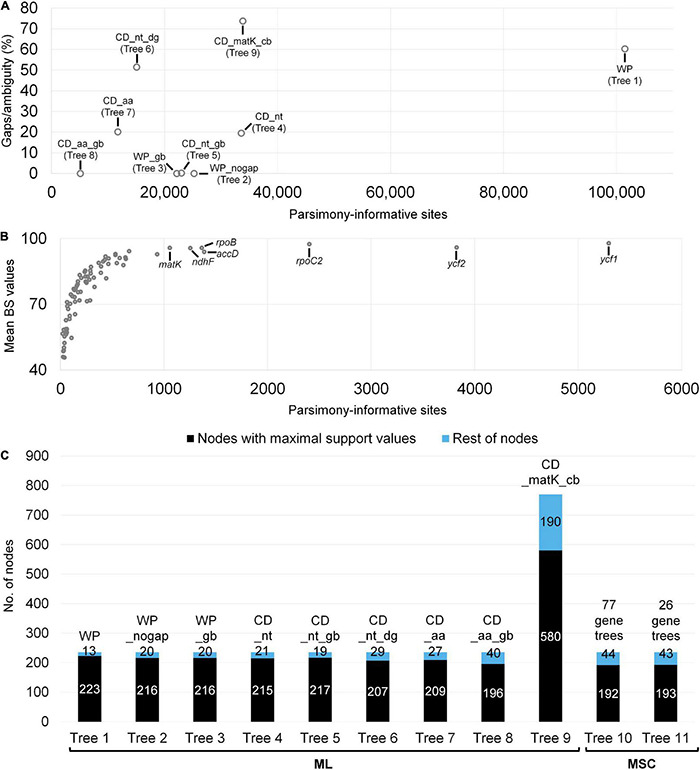
Comparison of datasets used for phylogenetic analyses and their resolution in resulting trees. Descriptions for these datasets provided in Table 1. **(A)** A scatter plot showing differences in number of parsimony-informative sites (PIS) and gap/ambiguity among datasets for maximum likelihood (ML) analysis. **(B)** A scatter plot showing PIS and mean bootstrap values (BS) of 77 individual gene trees that were used for the multispecies coalescent (MSC) approach. The top seven protein-coding regions (including *matK*) with PIS (>1,000) are indicated. **(C)** A comparison of tree topology resolutions. Values within the histograms indicate the number of nodes. Maximal support values are BS = 100 or local posterior probability = 1.0.

In total, 39 papilionoid plastomes were assembled and included in our analysis (Supplementary Table 1). Plastomes varied from 123,013 (*Astragalus canadensis* var. *brevidens*) to 168,148 bp (*Myrospermum sousanum* A.Delgado & M. C.Johnst.) in unit length. The read depth varied from 409 to 6,213×. Together with previously sequenced plastid data, eight plastome-only and one combined (*matK*-only + CD_nt) datasets were prepared for our ML analyses of papilionoid legumes (Tables 1 and Supplementary Table 3). The nine datasets for ML analyses varied in the values for the alignment length, proportion of gaps/ambiguities, and number of parsimony-informative sites (PIS) (Figure 2A). The highest values of the alignment length and PIS were shown in the WP dataset with 313,335 bp (60.3% gaps/ambiguities) and 101,445 sites, respectively, values substantially higher than the rest of the datasets. The original concatenation of all CDS (CD_nt) showed higher values for the alignment length and number of PIS, but a trimmed version (CD_nt_gb) was similar to plastome alignments without indel regions (WP_nogap) or with only conserved sequence blocks (WP_gb). Both modification of CD_nt (CD_nt_dg) and the combination of CD_nt with the *matK*-only dataset (CD_matK_cb) resulted in changes in the number of the PIS and gaps/ambiguity (Supplementary Table 3).

The datasets based on translated AAs showed the lowest values for alignment length and number of PIS. Datasets 10 and 11 (77 gene trees and 26 gene trees) employed for the MSC approach were prepared based on ML analyses for each individual CDS alignment (Supplementary Table 4). Length of each alignment varied from 90 bp (*petN*) to 11,175 (*ycf1*) bp. The lowest and highest mean BS values were shown from *psbN* (45.7) and ycf1 (97.9) (Figure 2B and Supplementary Table 4). There were 26 trees with average BS > 85 (i.e., dataset 11).

Based on 11 datasets, 11 trees were inferred and compared (Figure 2C). Tree 1 (WP dataset) resolved the most papilionoid nodes (223 out of 236, 93.5%). Trees 2—5 resolved a similar number of nodes with maximal support (BS = 100) (215—217, 91.1—91.9%). Trees 6—8 showed the lowest number of resolved nodes with maximal support (196—207, 83.1—87.7%) among ML trees. The many additional papilionoid nodes in Tree 9 (CDS and *matK*-only) resolved 580 out of 770 nodes (75.3%) with maximal support. Multispecies coalescence trees (10 and 11) resolved a similar number of nodes [192 (81.4%) and 193 (81.8%), respectively] with maximal support (LPP = 1.0).

**FIGURE 3:**
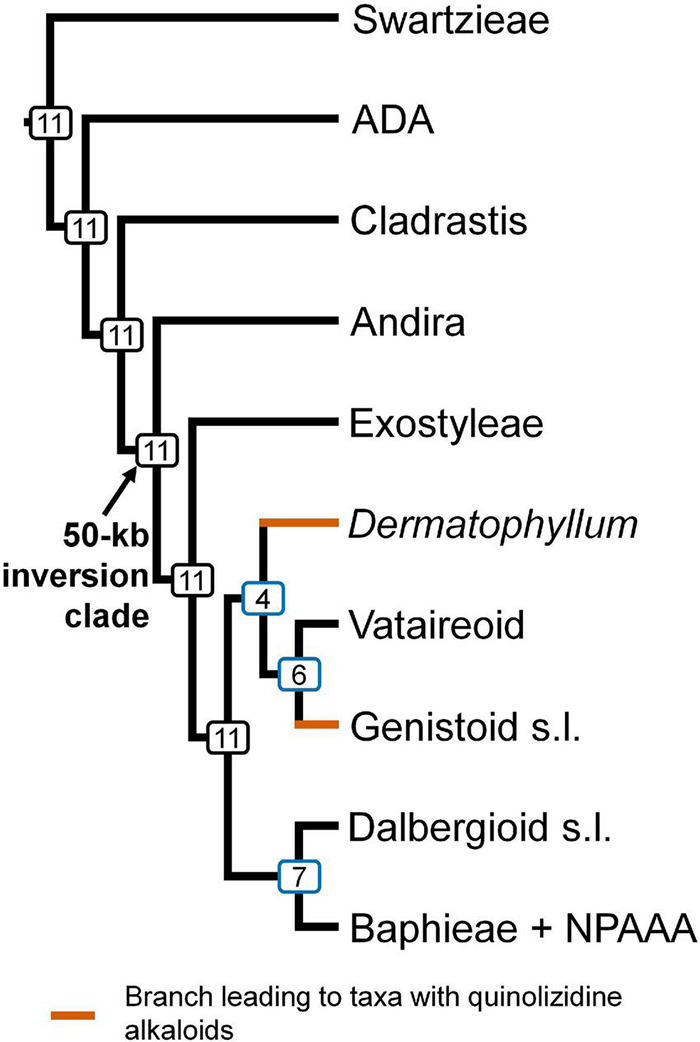
The consensus of 11 phylogenetic trees of papilionoid legumes based on analyses of plastome-scale and *matK* sequence data. The number of topologies in which this clade was recovered in 11 trees is indicated at each node. Branches leading to taxa known to produce quinolizidine alkaloids are highlighted in brown. NPAAA: non-proteinogenic amino acid accumulating, ADA: Angylocalyceae, Dipterygeae, and Amburaneae, kb: kilobase, s.l.: sensu lato.

**FIGURE 4:**
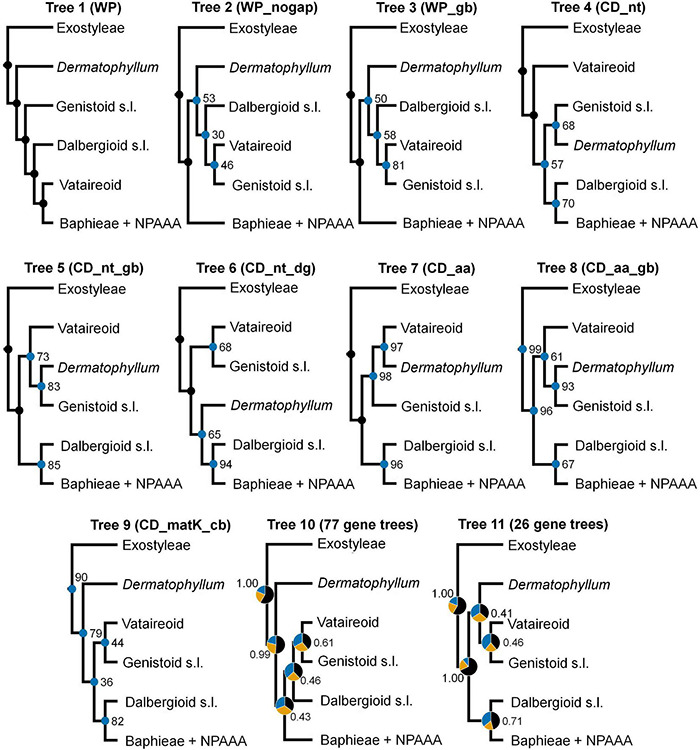
Differences in topology and support values for the 11 trees with respect to resolving the phylogenetic relationships among the Vataireoid, *Dermatophyllum*, Genistoid s.l., Dalbergioid s.l., and Baphieae + NPAAA (non-proteinogenic amino acid accumulating) clades. Nodes with maximal bootstrap support (100) are denoted as black dots, while other nodes are marked with blue dots with values in nine trees (Trees 1–9). In Trees 10 and 11, based on the multispecies coalescence approach, pie charts representing quartet score values of topologies (black = main, orange = first alternative, blue = second alternative) and local posterior probability are presented at nodes.

**FIGURE 5:**
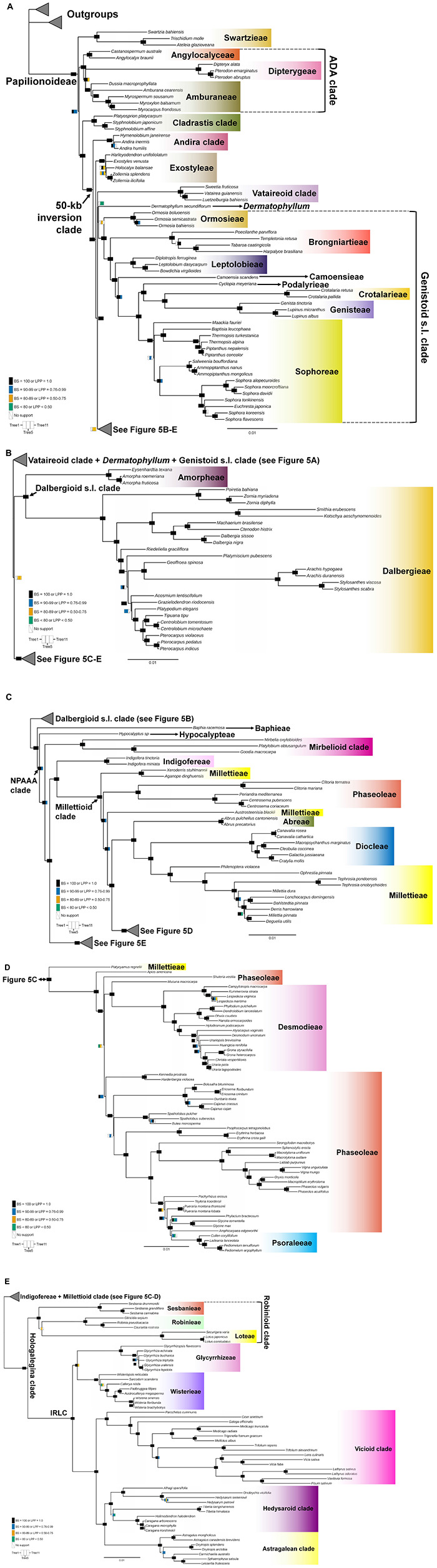
A maximum-likelihood tree based on a concatenated dataset of 77 plastome coding regions with Gblocks trimming (See Table 1). The figure is separated into five panels with a focus on the major clades. **(A)** Swartzieae, ADA (Angylocalyceae, Dipterygeae, and Amburaneae), Cladrastis, Andira, Exostyleae, Vataireoid, and Genistoid s.l. clades; **(B)** Dalbergioid s.l. clade; **(C)** Baphieae, Hypocalypteae, Mirbelioid, Indigofereae, Millettioid (part 1) clades; **(D)** Millettioid (part 2) clade and **(E)** Robinioid clade and inverted repeat-lacking clade (IRLC). Supporting values of three trees are visualized at nodes as rectangles. The rectangles are subdivided into three columns that correspond to Tree 1 (left, WP), Tree 5 (middle, CD_nt_gb), and Tree 11 (right, 26 gene trees). Colors in each column represent support values. Black [bootstrap support value (BS) = 100 or local posterior probability (LPP) = 1], Blue [BS (90–99) or LPP (0.76–0.99)], Orange [BS (80–89) or LPP (0.50–0.75)], Bluish green (BS < 80 or LPP 0.50). Scale indicates number of nucleotide substitutions per site. The colors for clades are not related to their support values. We generally followed the color scheme for early-branching papilionoid clades of Figures 2–5 in Cardoso et al. (2013a).

**FIGURE 6:**
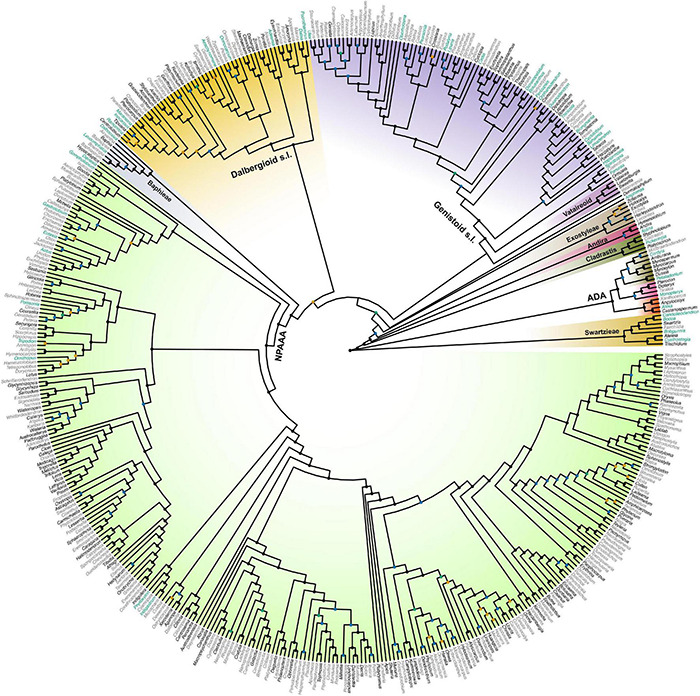
A genus-level phylogeny of Papilionoideae as produced by the maximum likelihood analysis of the dataset 9 (CD_matK_cb), which combined genera with fully sequenced plastomes and the expanded sampling of genera with *matK* sequence data only. Black nodes are supported by bootstrap values (BS) = 100 and blue nodes by BS = 90–99, while orange nodes are supported by BS = 80–89 and cyan nodes by BS = 70–79. Genera highlighted in black have already been fully sequenced for plastomes. The 50 genera highlighted in cyan are either still phylogenetically enigmatic, are morphological key genera, or represent monospecific lineages, all of which should be the focus of future plastome research. The colors for clades are not related to their support values. We followed the color scheme for papilionoid clades of Figure 1 in Cardoso et al. (2013a). NPAAA: non-proteinogenic amino acid accumulating, ADA: Angylocalyceae, Dipterygeae, and Amburaneae.

Topological concordance with regard to 10 main papilionoid lineages (Swartzieae, ADA, Cladrastis, Andira, Exostyleae, *Dermatophyllum*, Vataireoid, Genistoid s.l., Dalbergioid s.l., and Baphieae + NPAAA clades) was assessed across the 11 trees (Figure 3). Topologies showing the five lineages Swartzieae, ADA, Cladrastis, Andira, and Exostyleae clades as successive sister groups to a monophyletic group comprising the remaining five papilionoid lineages (*Dermatophyllum*, Vataireoid, Genistoid s.l., Dalbergioid s.l., and Baphieae + NPAAA clades) were consistently retrieved from all trees. The three nodes related to the five remaining lineages resolved differently. The most frequently recovered relationship subdivided the group into two clades of (Dalbergioid s.l., Baphieae + NPAAA) and (*Dermatophyllum*, (Vataireoid, Genistoid s.l.)). Support and alternative topologies for these five lineages are shown in Figure 4. While all 11 trees resolved relationships, only the nodes inferred in Tree 1 had 100% BS support. Among the seven ML trees (except Tree 1) based on plastome datasets, Trees 7 and 8 from translated AA alignments showed slightly better support for the interrelationships of the five lineages. The MSC-based Tree 10 had only low LPP and QS for the main topology of the five lineages. The selection of 26 genes with high mean BS values as the input dataset (dataset 11) showed slightly increased LPP and QS for the monophyly of the five remaining lineages but does not substantially affect resolution of relationships among these lineages (Tree 11). To show specific relationships within and among the 10 key papilionoid lineages, Tree 5 along with support values of Trees 1 and 11 are presented as the main tree (Figure 5). In total, the 50 papilionoid genera that need further attention in plastid phylogenomics are highlighted in Figure 6 based on the most comprehensive taxon sampling (CD_matK_cb). These 50 genera are either still phylogenetically enigmatic, are morphological key genera, or represent monospecific lineages.

## Discussion

### Plastid Phylogenomic Signals Across the Papilionoid Legumes

Maximum Likelihood analysis of the whole plastome alignment (Figure 4) resolved all deep relationships among major early-diverging clades of the Papilionoideae with maximal support values. This approach for phylogenetic analyses using the entire plastome as a single locus was rigorously tested in Poaceae (Duvall et al., 2020; Orton et al., 2021). Duvall et al. (2020) demonstrated how tree topology and support values could vary according to the level of gap removal from the whole plastome alignment, and recommended excluding any gapped positions from machine-generated alignments. Apart from concerns as to whether it is reasonable to recognize the plastome as a single locus (Gonçalves et al., 2020; Doyle, 2021), one of the main and practical criticisms about whole plastome alignment without further filtration is that most gaps are located in AT-rich and low complexity regions, which are prone to high-level length polymorphisms in simple sequence repeats and falsely aligned non-orthologous sequences (Duvall et al., 2020). The much longer whole plastome alignment of 244 legumes (313,335 bp) compared to the alignment lacking indels (64,099 bp) is not surprising when considering the dynamic gene, intron, intergenic, and repeat content across papilionoids (e.g., Milligan et al., 1989; Doyle et al., 1995; Bailey et al., 1997; Cai et al., 2008; Jansen et al., 2008; Sabir et al., 2014; Sveinsson and Cronk, 2014; Schwarz et al., 2015; Choi et al., 2019, 2020a; Jin et al., 2019; Oyebanji et al., 2020; Lee et al., 2021). The WP alignment extended well beyond the length of a typical plastome to accommodate for poorly aligned sequences. While the lability of papilionoid plastome intergenic regions may provide a much greater number of PIS, their inclusion can introduce error in the alignment and infer spurious relationships.

In the remaining ML trees based on datasets without highly divergent intergenic regions, three controversial nodes connected by very short branches were revealed within a monophyletic group that included the Vataireoid, *Dermatophyllum*, Genistoid s.l., Dalbergioid s.l., and Baphieae + NPAAA clades (see Figures 3–5). The trees that were derived from analyses of translated AA sequences showed slightly better support. Even though MSC methods were suggested as an alternative way to explore plastome data (Gonçalves et al., 2019, 2020), this approach also left problematic nodes with low support values and showed a high frequency of alternative tree topologies. The source of conflicting signals among plastid loci can be diverse, including systematic and stochastic errors (Walker et al., 2019). The vast majority of plastid genes are short (see Figure 2B and Supplementary Table 4) and often yield poorly resolved individual gene trees as input for the MSC approach (Walker et al., 2019; Doyle, 2021). The species tree estimated from the trees based on the highly informative 26 CDS loci that exhibited strong phylogenetic signals also showed a similar level of alternative topologies (Figure 4). A likely explanation for this phylogenetic uncertainty is that the time interval between the divergences was too short to achieve resolution even with such phylogenetically informative loci. This makes it challenging to avoid stochastic errors in a zone of rapid divergence at deep nodes, such as the case within the 50-kb inversion clade of the papilionoid phylogeny. Thus, comparison with a phylogeny based on other genomic data (e.g., Zhao et al., 2021) would be desirable.

So far, several plastid phylogenomic analyses at several taxonomic levels of Fabaceae have been conducted (LPWG, 2017; Koenen et al., 2020a; Oyebanji et al., 2020; Zhang et al., 2020; Aecyo et al., 2021), but none of these has paid particular attention to increasing taxonomic diversity to fully resolve the deep nodes of Papilionoideae. Our taxon sampling included one to several representatives of most evolutionary key lineages except for a few small groups (e.g., Cardoso et al., 2013a; LPWG, 2017). In the following sections, phylogenetic relationships of papilionoid lineages will be discussed in a systematic context with other relevant molecular and non-molecular evidence. Overall, our analyses have provided well-resolved and strongly supported phylogenies, which are in agreement with topologies retrieved from previous plastid sequence-based phylogenies, and essentially validate the resolving power of *matK* sequences alone for reconstructing papilionoid phylogenies (e.g., Hu et al., 2000; Lavin et al., 2001; McMahon and Hufford, 2004; Cardoso et al., 2013a). Compared to a recent nuclear phylogenomic study (Zhao et al., 2021), there were several notable relationships with various nodal support values that were not retrieved from plastid data alone. Sexual recombination is restricted or absent in wild-type plastomes, suggesting possible involvement of reticulation/introgression events in the formation of some ancestral papilionoid lineages.

### Swartzieae, ADA, and Cladrastis clades

The phylogenetic analyses in this study consistently resolved the early-diverging Swartzieae, ADA (Angylocalyceae, Dipterygeae, and Amburaneae), and Cladrastis clades, which collectively form a grade to the remaining Papilionoideae. Most genera within this grade were previously treated as members of tribes Sophoreae and Swartzieae by Polhill (1981b, 1994). In that sense, the grade comprising the Swartzieae, ADA, and Cladrastis clades, is reminiscent of Polhill’s (1981b, Figure 4; 1994) traditional “supposed relationship of tribes,” which recognized several groups of caesalpinioid-like genera among the largely tropical and subtropical Swartzieae and Sophoreae as transitional between subfamilies Caesalpinioideae and Papilionoideae. This group was considered to form a basal assemblage of the subfamily leading to the more derived papilionoid tribes. The taxonomic composition of this grade was partly recognized from early surveys for the absence/presence of a 50-kb inversion in legume plastomes (Doyle et al., 1996) and nodulation ability (Sprent, 2000).

Molecular phylogenetic analyses to date (Cardoso et al., 2012a, 2015; Ramos et al., 2016; LPWG, 2017; Zhang et al., 2020; Zhao et al., 2021) have reached a consensus that there are three main monophyletic groups that diverged early at the base of Papilionoideae. Early plastid sequence-based phylogenetic studies (Doyle et al., 1996, 1997; Pennington et al., 2001; Wojciechowski et al., 2004) suggested that groups of certain genera from the Swartzieae, Sophoreae, and Dipterygeae of Polhill’s traditional classification (Polhill, 1981b, 1994) were outside of the 50-kb inversion clade in Papilionoideae. A Swartzieae-derived monophyletic group as the first diverging lineage sister to all Papilionoideae was initially recognized by Pennington et al. (2001), while Doyle et al. (1997) and Wojciechowski et al. (2004) showed groups of additional genera from these tribes among the early-diverging clades. A sister relationship between a clade comprising the genera *Cladrastis* Raf., *Pickeringia* Nutt. ex Torr. & A.Gray, and *Styphnolobium* Schott, and the more derived 50-kb inversion clade, was initially revealed by Wojciechowski et al. (2004). Subsequent studies by Cardoso et al. (2012a; 2013a; 2015), Wojciechowski (2013a), and Duan et al. (2019), with more comprehensive taxon sampling based upon analyses of both plastid *matK* and nuclear rDNA regions, further clarified the composition of the Swartzieae, ADA, and Cladrastis clades. Relationships among these three clades remain uncertain. Recent plastome-based phylogenetic analyses employing multi-locus (Ramos et al., 2016) and complete plastome (supported by all 11 trees, see Figure 3; Zhang et al., 2020) datasets retrieved the (Swartzieae,(ADA,(Cladrastis,50-kb inversion clade))) topology while a recent nuclear phylogenomic study recovered the (ADA(Swartzieae,(Cladrastis,50-kb inversion clade))) topology (Zhao et al., 2021).

Given these results, the phylogenetic relationship of the Swartzieae and ADA clades remain equivocal. Both clades are highly diverse in floral structure (Figure 1) where almost all constituent genera display a particular floral architecture (e.g., Tucker, 1993, 2003b; Cardoso et al., 2013a; Leite et al., 2014, 2015; Prenner et al., 2015; Sinjushin, 2018). In addition to being distinguished by bilaterally or radially symmetrical apetalous flowers with a profusion of free stamens (e.g., *Cordyla*, *Swartzia* Schreb.; Figure 1A) or by single-petal flowers (e.g., *Amburana* Schwacke and Taub., *Swartzia*, *Trischidium* Tul.), representatives in these clades may have tiny radially symmetrical flowers measuring up to 3 mm long (*Myrocarpus* Allem., Figure 1D) to hardy, bat-pollinated flowers larger than 10 cm long (e.g., *Alexa* Moq.). Also, these clades include representatives with bilaterally symmetrical papilionate flowers that are unique in the Papilionoideae, either because of the wing-like, enlarged calyx lobes (e.g., *Dipteryx* Schreb., *Pterodon* Vogel; Figure 1C) or the fimbriate-glandular wing petals (e.g., *Petaladenium* Ducke). Such dramatic floral diversity in the early stages of the diversification history of the Papilionoideae indicates that resolving the relationships of the Swartzieae and ADA clades is fundamental, not just to better reconstruct the most likely ancestral form of the flower of the subfamily, but also to understand why their evolutionary history has been marked by profound deviations in flower morphology, from the typical papilionate architecture (as exhibited by those in Figures 1F,I–K,M–O).

### Meso-Papilionoideae (50-kb Inversion Clade)

Large inversions in the plastome were first identified in legumes (Palmer and Thompson, 1982; Bruneau et al., 1990) and subsequently in other plant groups (e.g., Asteraceae, Jansen and Palmer, 1987; Poaceae, Doyle et al., 1992). Early evidence for a monophyletic group of papilionoids marked by this apparently unique, derived synapomorphy (Doyle et al., 1996) has been confirmed by subsequent molecular phylogenetic studies (e.g., Doyle et al., 1997; Pennington et al., 2001; Wojciechowski et al., 2004). The presence/absence of the 50-kb inversion in sampled taxa (Doyle et al., 1996) not only clarified the positions of several generic groups of both Sophoreae and Swartzieae at the base of papilionoids or among more derived groups (Polhill, 1981b), but also revealed the existence of a large monophyletic group of higher papilionoids defined by this molecular synapomorphy that includes the vast majority of the subfamily. Indeed, this large core papilionoid group comprises 98% of Papilionoideae based upon current species diversity estimates (LPWG, 2021). With this fact in mind, and consistent with the principles of phylogenetic nomenclature (Cantino and de Queiroz, 2006; Cantino et al., 2007), we suggest the adoption of “Meso-Papilionoideae” for the 50-kb inversion clade as defined previously by Wojciechowski (2013b). The 50-kb inversion serves as a synapomorphy for this clade, supported by nuclear (Zhao et al., 2021), mitochondrial (Choi et al., 2021), and plastid (supported by all 11 trees, see Figure 3; Zhang et al., 2020) phylogenomic studies.

The recent discovery of three species of *Sesbania*, nested within the Hologalegina clade (Robinioid + IRLC; Figure 5; Wojciechowski et al., 2000), with plastomes that appear to have completely reverted the 50-kb inversion (Lee et al., 2021) does not diminish the phylogenetic significance of this large inversion early in papilionoids. The phylogenetic distribution of this plastome structure within *Sesbania* or in its close relatives in the Robinioid clade remains elusive.

Meso-Papilionoideae includes the species-rich Genistoid s.l., Dalbergioid s.l., and Baphieae + NPAAA clades, the small Andira, Exostyleae and Vataireoid clades, as well as the phylogenetically unresolved genera *Dermatophyllum* and the African *Amphimas*. Relationships among and within these clades will be discussed below except for *Amphimas*, from which complete plastome data is not yet available, but which has been placed in the 50-kb inversion clade based on *matK* sequence data (Figure 6; Cardoso et al., 2015).

### Andira and Exostyleae Clades

In our study, the Andira and Exostyleae clades were consistently (supported by all 11 trees, see Figure 3) resolved as successive sister groups to the monophyletic group comprising the remainder of the Meso-Papilionoideae (maximal support values from all three trees, see Figure 5A). The plastid phylogenomic study of Zhang et al. (2020), which included one taxon from each clade, also inferred the same topology. However, nuclear phylogenomic analysis (Zhao et al., 2021) placed *Andira inermis* (W. Wright) Kunth ex DC. sister to a clade of Baphieae + NPAAA, while Exostyleae was resolved as the first diverging lineage within Meso-Papilionoideae with maximum support values in all seven coalescent trees. The Andira clade (sensu Ramos et al., 2016), which includes three genera (*Aldina*, *Andira* Lam., and *Hymenolobium* Benth.), is expanded from Cardoso et al. (2012a, 2013a) to include *Aldina* based primarily on plastid data with some morphological similarities and shared ecological and distributional preference for Amazonian tropical rain forests. The Andira clade contains a mixture of taxa with radially symmetrical flowers with a profusion of exposed free stamens (*Aldina*) (see Figure 1D) and truly papilionate floral architecture (*Andira* and *Hymenolobium*). This was interpreted as an additional example of the common, interlaced phylogenetic distribution of the heterogeneous floral morphologies in early-diverging clades of Papilionoideae (Ramos et al., 2016). Whether the unexpected, more derived placement of *Andira* in Zhao et al. (2021) represents a signature of incongruence between plastid and nuclear genomes deserves further investigation.

### Genistoid s.l. and *Dermatophyllum* Relationships, and Implications for the Evolutionary Distribution of Quinolizidine Alkaloids in Legumes

The sister relationship between the Genistoid s.l. and *Dermatophyllum* lineages was retrieved from three ML analyses, where the highest support value (BS = 93) was found in Tree 8 (Figure 4). However, this relationship is equivocal because of conflicting alternative ML topologies based on analyses of the datasets derived from complete plastomes. In addition, the MSC tree estimations returned weakly supported relationships for these groups and inferred a high frequency of alternative topologies among individual CDS phylogenies.

Among legumes, QA production is restricted to most genistoid genera and to *Dermatophyllum* (Bisby et al., 1994; Kite and Pennington, 2003; Lee et al., 2013; Wink, 2013). This chemotaxonomic evidence has led to alternative hypotheses with respect to the controversial phylogenetic position of *Dermatophyllum*. One hypothesis suggests that the production of QAs is a derived characteristic defining a clade that includes the most recent common ancestor (MRCA) of the Genistoid s.l. and *Dermatophyllum* lineages, and thus forms strong evidence for a sister relationship of these two taxa (e.g., Cardoso et al., 2012a, 2013a; Kite et al., 2013; Lee et al., 2013). Alternatively, the genetic capacity for QA biosynthesis was established in the very early diversification of papilionoids but the genes remain silent (or were lost) in many descendant lineages (e.g., Wink et al., 2010). The Genistoid s.l. clade, as delimited in the phylogenetic analysis of *matK* sequences by Wojciechowski et al. (2004), included all known QA-accumulating genera except *Dermatophyllum* (as syn. *Calia* Terán and Berland.) and *Ormosia* Jacks. The Genistoid s.l. clade was subsequently expanded to accommodate the genus *Ormosia* and related genera of the Ormosieae, which has been consistently resolved as sister to the rest of the clade (Cardoso et al., 2012a, 2013a), suggesting that the only remaining QA-producing genus *Dermatophyllum* was likely sister to the Genistoid s.l. clade. The scenario of Wink et al. (2010) was based on an *rbcL* gene phylogeny that showed an early divergence of *Dermatophyllum* within a monophyletic group containing *Myroxylon* L. (Amburaneae) that does not produce QAs and is not a member of Meso-Papilionoideae. However, our study (Figures 3–5) and others (Zhang et al., 2020) with broad taxon sampling of papilionoid legumes resolved *Dermatophyllum* as a more derived lineage in the Meso-Papilionoideae. Wink et al. (2010) suggested that QA production was one of many herbivore defense strategies rendering it dispensable, as exemplified by loss or extreme reduction of QA production in some species within genistoid genera such as *Crotalaria* L., *Lotononis* (DC.) Eckl. and Zeyh., *Ulex* L., *Calicotome* Link, and *Spartocytisus* Webb and Berthel.

In light of our results, modified ancient gain and loss scenarios regarding QA production can be postulated according to the alternative phylogenetic positions of *Dermatophyllum* (supported by BS value > 90) relative to the Genistoid s.l., Vataireoid, Dalbergioid s.l., and Baphieae + NPAAA clades. One scenario posits that the QA biosynthesis pathway was present at least since the MRCA of all five lineages but was lost after the successive divergence of the *Dermatophyllum* and the Genistoid s.l. clades (Figure 4, Tree 1). Alternatively, QA production is ancestral in only three lineages (*Dermatophyllum*, Genistoid s.l. and Vataireoid clades) but was lost early in the diversification of the Vataireoid clade. Our study does not confidently identify the position of *Dermatophyllum*, but it reduces the number of possible solutions by resolving other early-diverging relationships within Meso-Papilionoideae. Nevertheless, the *Dermatophyllum*-Genistoid s.l. sister hypothesis remains the most parsimonious (single-step, excluding recent loss events in the genistoids) scenario. In order to shed light on the evolutionary pathway(s) leading to the biosynthesis of QAs, resolving the relationship of *Dermatophyllum* with respect to the remaining genistoids is a high priority in future nuclear-based phylogenomics of the early-diverging Papilionoideae.

### Vataireoid Clade

The exclusively neotropical Vataireoid clade includes just 28 species, and is another example of an early-diverging papilionoid lineage outside the large NPAAA clade with heterogeneous floral morphology (Cardoso et al., 2013b), similar to the Andira clade (sensu Ramos et al., 2016). Monophyly of this morphologically heterogeneous group was highly supported in phylogenetic analyses based on single plastid genes or a few combined nuclear and plastid genes (Cardoso et al., 2012a, 2013a,b) to genome-scale data (Figure 5A; Zhao et al., 2021). There was no strongly supported sister relationship hypothesis for this clade except Zhao et al. (2021). The close affinity of the vataireoids to Dalbergieae based on a single-seeded samaroid fruit morphology was suggested by Lima (1980; 1982; 1990), but molecular systematic studies have not supported this relationship. The phylogenetic position of the clade is inconclusive based on our analyses, but a nuclear phylogenomic study (Zhao et al., 2021) resolved the clade as sister to a monophyletic group (including Dalbergioid s.l., Genistoid s.l., Andira, and Baphieae + NPAAA clades) that includes all known taxa with the ability to nodulate within Meso-Papilionoideae (Sprent et al., 2017; Ardley and Sprent, 2021).

### Dalbergioid s.l. Clade

The Dalbergioid s.l. clade includes monophyletic groups of the pantropical Dalbergieae and the predominantly North American temperate Amorpheae (Wojciechowski et al., 2004). In our study, this clade most frequently grouped with the NPAAA + Baphieae clade, albeit with alternative positions and low support values (Figures 3, 4). A sister group relationship of the Andira clade with the Dalbergioid s.l. clade was once weakly supported (Wojciechowski et al., 2004), but further support for that relationship is lacking. A nuclear phylogenomic study (Zhao et al., 2021) grouped the Dalbergioid s.l. clade with the Genistoid s.l. clade but with weak support (highest BS value from seven coalescent trees was 77). As such, the sister relationship of the dalbergioids within Meso-Papilionoideae is still unclear.

While both nuclear phylogenomic (Zhao et al., 2021) and our plastid phylogenomic (Figures 3–5) analyses have failed to clarify the relationships of the Dalbergioid s.l. clade, they concur with previous comprehensively sampled *matK* phylogenies in strongly supporting the monophyly and interrelationships of three main subclades within the dalbergioids: the *Adesmia*, *Dalbergia*, and *Pterocarpus* clades (Lavin et al., 2001; Wojciechowski et al., 2004; Cardoso et al., 2012a, 2013a). Additionally, by resolving the radially symmetrical flowered genera *Riedeliella* Harms and *Acosmium* Schott in isolated positions within Dalbergieae, our analyses based on complete plastomes demonstrated yet again how the independent evolution of non-papilionate floral architecture has been so recurrent among the early-branching papilionoids (Lavin et al., 2001; Cardoso et al., 2012a,b).

### Baphieae and Non-protein Amino Acid Accumulating Clades

A single origin of non-protein amino acid biosynthesis in papilionoids was hypothesized by Bell (1981) because canavanine, a close analog of arginine, was almost mutually exclusive of alkaloid accumulation, and restricted to 16 closely related papilionoid tribes. A monophyletic group containing all known taxa producing non-protein amino acids, the NPAAA clade (Cardoso et al., 2012b), is supported by all recent phylogenetic analyses (LPWG, 2017; Koenen et al., 2020b, 2021; Zhang et al., 2020; Choi et al., 2021; Zhao et al., 2021). This clade includes several of the most species-rich (ca. 11,000 spp.) and rapidly evolving legume lineages (Cardoso et al., 2013a; Koenen et al., 2013), as well as the largest flowering plant genus *Astragalus* L. (ca. 3000 spp.) and the most agriculturally important culinary pulses such as common beans, peas, lentils, and soybeans (Gepts et al., 2005). The clade also stands out among the main Papilionoideae lineages with respect to the almost universal evolutionary canalization of the specialized, strongly bilaterally symmetrical papilionate floral architecture. Thus far, there are no examples of reversion to radial floral symmetry or profusion of free stamens as found in earlier branching clades (Lewis et al., 2005; Cardoso et al., 2013a).

The NPAAA clade is sister to the small (c. 60 spp.), predominantly African Baphieae clade, which contains genera with both radially and bilaterally symmetrical flowers. Evolutionary transitions between polysymmetry and monosymmetry in floral architecture are common in angiosperms (Endress, 1996, 1999), such that the emergence of the core eudicots, a clade with more than 200,000 species, coincides with the fixation of pentamerous flowers, whorl organization, and a perianth often differentiated into sepals and petals (Specht and Bartlett, 2009). Such changes in floral architecture may have led to the recurrent evolution of bilateral symmetry (zygomorphy) from polysymmetric-flowered ancestors across angiosperms. This shift coincides with the co-diversification of megadiverse families and specialized pollinating insects (Cardinal and Danforth, 2013). These factors may have contributed to increased speciation rates (Sargent, 2004; Davis et al., 2014) and monosymmetry as a key innovation during the radiation of angiosperms (Sanderson and Donoghue, 1994; Bond and Opell, 1998). Likewise, the evolutionary maintenance of the papilionate flower may have sparked the explosive diversification of the large NPAAA clade, which includes almost 70% of both specific and generic diversity in Papilionoideae (Cardoso et al., 2012a, 2013b).

Here, two large clades within the NPAAA clade have been confirmed in agreement with all previous molecular phylogenetic studies (Figures 5C–E; Wojciechowski et al., 2004; Cardoso et al., 2013a; Queiroz et al., 2015; LPWG, 2017; Zhao et al., 2021): the Indigofereae + Millettioid and Hologalegina (Robinioid + IRLC) clades. Most of the relationships among the main lineages within the Millettioid clade concur with previous studies (Figures 5C,D; Queiroz et al., 2015; Zhao et al., 2021), however, a discordance was observed in the sister relationship of *Mucuna* Adans. (Phaseoleae) and Desmodieae. *Mucuna* was supported as the sister to Desmodieae in plastid phylogenies (Doyle et al., 2000; Kajita et al., 2001; Stefanović et al., 2009; Jin et al., 2019). This sister relationship is also marked by the shared loss of the plastid *rpl2* intron, which appears to have been lost only a few times in legumes (Doyle et al., 1995; Bailey et al., 1997; Lai et al., 1997; Jin et al., 2019). While our analyses of complete plastome data showed *Mucuna* as sister (maximal support values from all three trees, see Figure 5D), or part of a sister clade (including *Craspedolobium* Harms and *Haymondia* A.N.Egan & B.Pan) to Desmodieae (Figure 6), Zhao et al.’s (2021) nuclear phylogenomic analyses resolved *Mucuna* as sister to *Cochlianthus* Benth., combining a Mucuna-Cochlianthus clade as sister to *Apios* Fabr., whereas *Craspedolobium* and *Haymondia* were successive sister groups of Desmodieae. Lackey (1981) considered the genera *Apios*, *Cochlianthus*, and *Mucuna* to be members of an artificial amalgamation that defined the Phaseoleae subtribe Erythrininae, but considered *Apios* and *Cochlianthus* to be a natural grouping or even congeneric, making the sister relationship between *Cochlianthus* and *Mucuna* largely unexpected. However, the key feature of *Haymondia* (i.e., an explosive flower tripping mechanism involving the upward movement of the reproductive column that remains fully reflexed from the wing and keel petals, and touching the standard petal; Egan and Pan, 2015) can also be found in some genera of Phaseoleae (*Apios*, *Cochlianthus*, and *Mucuna*) and Desmodieae (except the Lespedeza group) within the millettioids (Schrire et al., 2009). Due to its phylogenetically scattered distribution that includes Indigofereae and *Medicago* L. within the NPAAA clade, as well as in the genistoids *Genista* L., *Harpalyce* Moc. and Sessé ex DC., *Spartium* L., and *Ulex* L., and the dalbergioid *Brya* P. Browne (Arroyo, 1981; Lavin et al., 2001; Schrire et al., 2009), convergent evolution of this pollination-related morphological feature cannot be ruled out. The presence of a cryptic, shared morphological feature between two distantly related clades, including *Apios* and Desmodieae and the highly supported, yet conflicting positions of *Mucuna* in nuclear and plastid phylogenies supports putative ancestral hybridization in these groups (Egan et al., 2016).

The Hologalegina clade was first designated by Wojciechowski et al. (2000) to comprise the traditionally recognized “temperate herbaceous tribes” of Polhill (1981b, 1994). This large monophyletic group includes two main, well supported subclades; Robinoid (Sesbanieae, Loteae, and Robinieae) and the IRLC. Even though some relationships within the Robinioid clade and IRLC need further clarification, the monophyly of each group has been consistently supported in plastid-based phylogenies (maximal support values from all three trees, see Figure 5E; Wojciechowski et al., 2004; Lavin et al., 2005). The nuclear phylogenomics study of Zhao et al. (2021) did not support the monophyly of the Robinioids and resolved Sesbanieae + Loteae as sister to the IRLC, albeit with low support values (4 out of 7 trees > BS 70). Indeed, Loteae was once regarded as closer to members of the IRLC than to Robinieae, based on similarities in vegetative morphology, growth habit, and distribution (Polhill, 1981b, 1994). Similarly, the early nuclear rDNA-ITS-based phylogeny of Hu et al. (2000) and recent mitogenome-based phylogeny of Choi et al. (2021), with limited taxon sampling (without Sesbanieae), resolves *Lotus* L. as sister to the IRLC.

Within the Vicioid clade (IRLC), five genera of Trifolieae (*Medicago*, *Melilotus* (L.) Mill., *Ononis* L., *Trifolium* L., *Trigonella* L., excluding *Parochetus* Buch.-Ham. ex D.Don), have been resolved as paraphyletic, placing *Trifolium* as sister to the monophyletic Fabeae in plastid phylogenies (Wojciechowski et al., 2000, 2004; Steele and Wojciechowski, 2003; Schaefer et al., 2012). Our study also showed the sister relationship of *Trifolium* to Fabeae (maximal support values from two of three trees, see Figure 5E). However, the nuclear phylogenomic study resolved *Trifolium* as sister to a clade composed of *Medicago*, *Melilotus*, and *Trigonella* with maximal support values from all seven coalescent trees (Zhao et al., 2021). Similarly, but with very limited sampling, a mitochondrial phylogenomic study (Choi et al., 2021) also supported a monophyletic group based on *Medicago* and *Trifolium* as sister to *Vicia* (Fabeae). This grouping of four Trifolieae genera (*Medicago*, *Melilotus*, *Trifolium*, and *Trigonella*) is also marked by the loss of mitochondrial *rps1* due to its functional, intracellular gene transfer to the nuclear genome (Hazle and Bonen, 2007; Choi et al., 2020b). In the case of Trifolieae, in which the nuclear and mitochondrial data produce topologies that agree closely with a classification based on gross morphology while the plastid data does not, a plastid capture scenario is worth considering.

Phylogenies based on plastid and mitochondrial genomes can produce different topologies for a lineage because of biparental organelle inheritance together with cytonuclear incompatibility, as exemplified in the IRLC genus *Pisum* L. (Bogdanova et al., 2021). Many IRLC taxa share the potential for biparental plastid inheritance (Corriveau and Coleman, 1988; Zhang et al., 2003). That a limited number of taxa have been tested for potential paternal transmission of organelle genomes and that the mitogenome-based phylogeny showed an alternative topology with regard to *Lotus* and *Trifolium* (Choi et al., 2021) warrants further investigation on the mode of organelle inheritance in Hologalegina. Within the IRLC, plastid capture scenarios have been suggested for various lineages based on conflicting results between nuclear rDNA and plastid data-based phylogenies (e.g., Ellison et al., 2006; Duan et al., 2021). Discordant taxon sampling across the three genomic datasets and the topological conflicts, which may have originated from the complex evolutionary behavior of repetitive nuclear rDNA (Álvarez and Wendel, 2003), hinder the detailed examination of those scenarios.

### Future Research Directions in Plastid Phylogenomics of the Papilionoid Legumes

Our study provides well resolved and strongly supported plastid phylogenies for the papilionoid legumes. Nevertheless, there remain nodes lacking phylogenetic resolution. The inclusion of non-CDS regions of plastomes could add more phylogenetic signal but at the potential expense of introducing a great amount of homoplasy, which could either decrease phylogenetic support or introduce bias toward unreliable relationships. There are further limitations on the use of plastome data despite that they have served well as a fundamental source of phylogenetic information. This information has helped us to address complex evolutionary issues in the papilionoid legumes, such as the origin of their extremely diverse floral architectures and secondary metabolites. The ancient, rapid diversification and reticulation/introgression detected in plastome-based Papilionoideae phylogenies warrant the incorporation of nuclear and mitochondrial data from concordant, broad taxon sampling. Perception and reconciling of conflicting phylogenetic signals within and between the three genomes are in their primary stage, and more complex evolutionary patterns may be revealed from the total genomic loci across all levels of subdivision in the Papilionoideae. In Hologalegina in particular, where the transition of maternal to biparental plastid inheritance as well as reticulation/introgression(s) likely occurred, more conflicts are expected.

Closing the sampling gaps within and between *matK*-only and complete plastome datasets could shed light on future sampling directions in Papilionoideae phylogenomics. Since Cardoso et al. (2013a) estimated the number of early-branching papilionoid genera that were not sampled for *matK* sequence data (52 out of the 196), there has been great progress in filling the sampling gap by virtue of individual locus or whole plastome sequences (e.g., Cardoso et al., 2015, 2017; Swanepoel et al., 2015; LPWG, 2017; Queiroz et al., 2017; Zhang et al., 2020). Our *matK*-only + plastome combined data set (Supplementary Table 5) includes 39 genera that were previously unsampled by Cardoso et al. (2013a), however, 12 genera (one is synonymized, see Supplementary Table 6) still remain to be fully sequenced. Moreover, many genera are still not well resolved in phylogenies based on *matK*-only data, such as the African tree genus *Amphimas* (Cardoso et al., 2012a, 2013a, 2015). Further plastome research should focus on examining the positions of phylogenetically unresolved genera, as well as those that are morphological key groups or represent monospecific lineages (Figure 6). Next generation sequencing applied to museomics (Bakker et al., 2016; Johnson et al., 2019) has proven to be a feasible approach to collecting genomic data from taxa for which fresh tissues are unavailable.

## Data Availability Statement

The datasets presented in this study can be found in online repositories. The names of the repository/repositories and accession number(s) can be found below: GenBank with accessions MZ725323, OL672849-OL672886. All sequence alignments and trees that were generated from this study are submitted to Dryad (https://doi.org/10.5061/dryad.sf7m0cg7m).

## Author Contributions

MW, TR, RJ, DC, and I-SC: conception and experimental design. MW, RJ, and TR: acquisition of funds. DC, RJ, TR, I-SC, HL, LQ, and MW: field collections. I-SC, CL, and TR: nucleic acid isolation. CL and I-SC: plastome assembly and annotation. I-SC, DC, and MW: DNA alignments and phylogenetic analyses and data interpretation. I-SC and DC: production of figures and tables. I-SC, DC, MW, TR, and RJ: writing and revision of the manuscript. All authors read and commented on the manuscript.

## Conflict of Interest

The authors declare that the research was conducted in the absence of any commercial or financial relationships that could be construed as a potential conflict of interest.

## Publisher’s Note

All claims expressed in this article are solely those of the authors and do not necessarily represent those of their affiliated organizations, or those of the publisher, the editors and the reviewers. Any product that may be evaluated in this article, or claim that may be made by its manufacturer, is not guaranteed or endorsed by the publisher.

## Footnotes

1
https://www.ncbi.nlm.nih.gov/genbank/

2
http://www.fao.org/pulses-2016/en/

3
https://datadryad.org

4
https://www.geneious.com

5
https://github.com/domingoscardoso/catGenes

6
http://www.phylotools.com/
