# Erratum: Highly Resolved Papilionoid Legume Phylogeny Based on Plastid Phylogenomics

**DOI:** 10.3389/fpls.2022.930260

**Published:** 2022-05-24

**Authors:** Frontiers Production Office

**Affiliations:** Frontiers Media SA, Lausanne, Switzerland

**Keywords:** deep evolution, Meso-Papilionoideae, plastid genome, Papilionoideae, Leguminosae

Due to a production error, the reference and citation for “DRYFLOR, 2016” was incorrectly written as “DRYFLOR et al., 2016” and “Dryflor, Banda-R. K., Delgado-Salinas, A., Dexter, K. G., Linares-Palomino, R., and Oliveira-Filho, A. (2016). Plant diversity patterns in neotropical dry forests and their conservation implications. *Science* 353, 1383–1387. doi: 10.1126/science.aaf508”. It should be “DRYFLOR, 2016” and “DRYFLOR (2016). Plant diversity patterns in neotropical dry forests and their conservation implications. *Science* 353, 1383–1387. doi: 10.1126/science.aaf5080”.

Due to a production error, the reference and citation for “LPWG, 2013” was incorrectly written as “LPWG et al., 2013” and “LPWG, Bruneau, A., Doyle, J. J., Herendeen, P., Hughes, C., and Kenicer, G. (2013). Legume phylogeny and classification in the 21st century: progress, prospects and lessons for other species-rich clades. *Taxon* 62, 217–248. doi: 10.12705/622.8”. It should be “LPWG, 2013” and “LPWG [Legume Phylogeny Working Group] (2013). Legume phylogeny and classification in the 21st century: Progress, prospects and lessons for other species-rich clades. *Taxon* 62, 217–248. doi: 10.12705/622.8”.

Due to a production error, the reference and citation for “LPWG, 2017” was incorrectly written as “LPWG et al., 2017” and LPWG, Azani, N., Babineau, M., Bailey, C. D., Banks, H., Barbosa, A. R., et al. (2017). A new subfamily classification of the Leguminosae based on a taxonomically comprehensive phylogeny. *Taxon* 66, 44–77. doi: 10.12705/661.3”. It should be “LPWG, 2017” and “LPWG [Legume Phylogeny Working Group] (2017). A new subfamily classification of the Leguminosae based on a taxonomically comprehensive phylogeny. *Taxon* 66, 44–77. doi: 10.12705/661.3”.

Due to a production error, the reference for “LPWG, 2021” was incorrectly written as “LPWG, Bruneau, A., Doyle, J. J., Herendeen, P., Hughes, C., and Kenicer, G. (2013). Legume phylogeny and classification in the 21st century: progress, prospects and lessons for other species-rich clades. Taxon 62, 217–248. doi: 10.12705/622.8”. It should be “LPWG, 2021. *The World Checklist of Vascular Plants (WCVP): Fabaceae, vers. June 2021*, ed R. Govaerts. Available online at: http://sftp.kew.org/pub/data_collaborations/Fabaceae/DwCA/.”

The publisher apologizes for these mistakes. The original version of this article has been updated.

## Publisher's Note

All claims expressed in this article are solely those of the authors and do not necessarily represent those of their affiliated organizations, or those of the publisher, the editors and the reviewers. Any product that may be evaluated in this article, or claim that may be made by its manufacturer, is not guaranteed or endorsed by the publisher.
